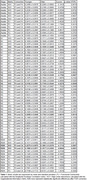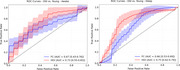# Age effects on brain network dynamics at different sleep stages

**DOI:** 10.1002/alz70856_107265

**Published:** 2026-01-08

**Authors:** Sebastian Moguilner, Shahab Haghayegh, Agustin Ibanez, Kun Hu

**Affiliations:** ^1^ Massachusetts General Hospital, Boston, MA, USA; ^2^ Latin American Brain Health Institute (BrainLat), Universidad Adolfo Ibañez, Santiago, Chile

## Abstract

**Background:**

Brain aging is associated with functional changes, including sleep dynamics as characterized by more wake‐up/fragmentations and less deep or REM (rapid‐eye‐movement) sleep. The aim of this study is to determine the age effects on brain network connectivity (BNC: interactions among different regions) at different sleep/wake stages.

**Method:**

We analyzed overnight average‐referenced 4‐channel EEG (F3, F4, C3, C4) from the Bitbrain Open Access Sleep database, including 84 young adults (<55 years; mean age = 29.61 ± 6.71[SD]) and 42 older adults (≥55 years; mean age = 67.19 ± 6.46). To assess BNC in each sleep/wake stage (wake, N1, N2, N3, REM), we obtained (1) the amplitude envelope correlation (AEC) that quantifies pairwise interaction or functional connectivity (FC) between each pair of two EEG channels; and (2) the O‐info metric in triplets of brain regions (2 channels vs the rest of the channels) — a higher‐order interaction (HOI) measure to quantify the redundancy and synergy of brain activities in multiple regions. Using logistic regression models with bootstrapped cross‐validation (5,000 iterations, 80% training and 20% testing), we also tested the performance of FC and HOI in distinguishing two groups.

**Result:**

Compared to young adults, the older adults had lower O‐info values during N1, N2, N3, and REM stages in interlobar and interhemispheric channels (all FDR‐corrected p values <0.05) (see Table 1). No group differences in AEC and O‐info were observed in the other channel‐stage combinations. Consistently, both measures could separate the two groups while O‐info appeared to have better performance during wakefulness (AUC = 0.73 [0.55‐0.83] for O‐info, and 0.67 [0.45‐0.76] for AEC) and during sleep (AUC = 0.75 [0.62‐0.79] for O‐info, and 0.66 [0.53‐0.69] for AEC) (see Figure 1).

**Conclusion:**

Decreased HOI values in older adults during sleep indicate altered brain network with loss or reduction of backup pathways, possibly reflecting reduced compensatory mechanisms with aging. The better performance of HOI in classifying age groups suggests that HOI may be more sensitive to age‐related brain network alterations. Future research is warranted to explore its application in early detection or prediction of neurodegenerative diseases.